# Expanding the BLUP alphabet for genomic prediction adaptable to the genetic architectures of complex traits

**DOI:** 10.1038/s41437-018-0075-0

**Published:** 2018-05-16

**Authors:** Jiabo Wang, Zhengkui Zhou, Zhe Zhang, Hui Li, Di Liu, Qin Zhang, Peter J. Bradbury, Edward S. Buckler, Zhiwu Zhang

**Affiliations:** 1 Department of Animal Science and Technology, Northeast Agricultural University, Harbin, China; 2 Institute of Animal Husbandry, Heilongjiang Academy of Agricultural Science, Harbin, China; 3 Department of Crop and Soil Sciences, Washington State University, Pullman, Washington USA; 4 Institute of Animal Science, Chinese Academy of Agricultural Sciences, Beijing, China; 5 Guangdong Provincial Key Lab of Agro-animal Genomics and Molecular Breeding, College of Animal Science, South China Agricultural University, Guangzhou, 510642 China; 6 Department of Animal Breeding and Genetics, College of Animal Science and Technology, China Agricultural University, Beijing, 100193 China; 7 United States Department of Agriculture – Agricultural Research Service, Ithaca, New York USA

## Abstract

Improvement of statistical methods is crucial for realizing the potential of increasingly dense genetic markers. Bayesian methods treat all markers as random effects, exhibit an advantage on dense markers, and offer the flexibility of using different priors. In contrast, genomic best linear unbiased prediction (gBLUP) is superior in computing speed, but only superior in prediction accuracy for extremely complex traits. Currently, the existing variety in the BLUP method is insufficient for adapting to new sequencing technologies and traits with different genetic architectures. In this study, we found two ways to change the kinship derivation in the BLUP method that improve prediction accuracy while maintaining the computational advantage. First, using the settlement under progressively exclusive relationship (SUPER) algorithm, we substituted all available markers with estimated quantitative trait nucleotides (QTNs) to derive kinship. Second, we compressed individuals into groups based on kinship, and then used the groups as random effects instead of individuals. The two methods were named as SUPER BLUP (sBLUP) and compressed BLUP (cBLUP). Analyses on both simulated and real data demonstrated that these two methods offer flexibility for evaluating a variety of traits, covering a broadened realm of genetic architectures. For traits controlled by small numbers of genes, sBLUP outperforms Bayesian LASSO (least absolute shrinkage and selection operator). For traits with low heritability, cBLUP outperforms both gBLUP and Bayesian LASSO methods. We implemented these new BLUP alphabet series methods in an R package, Genome Association and Prediction Integrated Tool (GAPIT), available at http://zzlab.net/GAPIT.

## Introduction

One of the ultimate goals of genomic research is to predict phenotypes from genotypes. This line of research is named genomic prediction. Genomic prediction in the human has the potential to lead to better medical treatments. For example, disability or the need for hip replacement surgery could be prevented if an increased risk of developmental dysplasia of the hip could be determined at a young age (Kurtz et al. 2007; Andersson and American Academyof Orthopaedic Surgeons 2015). Genomic prediction, also named as genomic selection in crops and livestock, has the potential to reduce breeding costs by eliminating individuals with less potential at an early stage (Heffner et al. 2011; Tempelman 2015; Wolc et al. 2016; Yu et al. 2016).

Revealing an individual’s genetic merit has enabled revolutionary changes in agricultural production. The productivity of most crops and livestock has doubled or tripled over the last 50 years (VanRaden et al. 2009). An individual’s genetic merit can be further leveraged by using detailed genomic information (de los Campos et al. 2010). With genetic markers that cover entire genomes, genetic prediction for a complex trait can be achieved at a very-early stage, that is, as soon as a DNA sample can be acquired (Wray et al. 2007; Guo et al. 2011). When evaluating a young bull, the information on a DNA chip—at a price of less than one hundred dollars—could save progeny tests on several hundred daughters and reach the same prediction accuracy (Hayes et al. 2010).

Prior to using genetic markers, the genetic merits of individuals were predicted directly as the best linear unbiased prediction (BLUP) using a fixed and random effects mixed linear model (MLM). Individuals’ total genetic effects were treated as random effects with variance structure defined by the pedigree-based kinship (Henderson 1984). This method played the most important role for the genetic improvement in livestock (Hayes et al. 2009a). Many statistical software packages have been developed to implement MLM, including those freely available for public use, such as multiple trait derivative free restricted maximum likelihood (MTDFREML) (Boldman et al. 1993). In the 1990s, more genetic markers became available. In 1994, Rex Bernardo introduced markers covering the whole maize genome to derive marker-based kinship among inbred lines and, in turn, genomic predictions for their genetic merits (Bernardo 1994, 1996).

Inspired by marker-assisted selection (MAS) developed in the late 20th century, all available genetic markers were fit simultaneously as random effects to avoid the model over-fitting problem in MAS (Meuwissen et al. 2001). The effects of all markers were then summed together to estimate an individual’s total genetic value. The random effects were assumed to have a normal distribution with variances following a certain prior distribution. Bayesian methods were employed for the posterior estimates of the random marker effects. When the variances are forced to be the same and a flat prior is applied to its prior distribution, the model is equivalent to ridge regression (RR). With different prior distributions, multiple Bayesian methods were developed, such as Bayes A, B, LASSO, and Cpi, creating a long series of analysis options, known as the Bayesian alphabet (Zhang 2004; Lorenzana and Bernardo 2009; Endelman 2011; Colombani et al. 2013). Among them, Bayesian LASSO is commonly used due to the widely available implementation of the LASSO algorithm (Park and Casella 2008).

In 2007, the pedigree-based kinship in widely used software packages, such as MTDFREML, was replaced by the marker-based kinship (Zhang et al. 2007). Soon after, a computationally efficient algorithm was developed to derive the marker-based kinship (VanRaden 2008). Due to the advantages of fast computing times and similarity to the existing models and software, this marker-based kinship approach—well-known as genomic BLUP or genomic best linear unbiased prediction (gBLUP)—quickly became the practical method for genomic selection (Hayes et al. 2009b). Furthermore, a single-step GBLUP (ssGBLUP) approach was developed for situations when simultaneous use of marker-based kinship and pedigree-based kinship is advantageous (Aguilar et al. 2010; Christensen and Lund 2010).

Similar to the development of the Bayesian alphabet series, many efforts have been conducted to enrich the BLUP methods by using different kinship-derivation algorithms. In fact, many of the kinship algorithms were developed before the introduction of gBLUP. One category of algorithms defines kinship as genetic similarity based on calculating genetic distances, such as Nel’s distance (Rousset 2000). Another category directly calculates kinship (Loiselle et al. 1995; Ritland 1996). Dedicated software packages have also been developed to derive kinship from markers, such as SPeGedi (Hardy and Vekemans 2002). Ultimately, however, improvement on gBLUP’s prediction accuracy was found using kinship derived from weighted markers. This method was named trait-specific relationship matrix (TA) BLUP (taBLUP) (Zhang et al. 2011). taBLUP conducts a genome-wide association study (GWAS) first and then uses the association signals to weight markers. Markers with stronger associations contribute more to kinship than markers with weaker associations. Simulations demonstrated that taBLUP had higher accuracy than gBLUP, but still lower accuracy than a Bayesian method (B) for traits controlled by smaller numbers of Quantitative Trait Nucleotides (QTNs) (Zhang et al. 2011).

Many studies have also been conducted to compare prediction accuracy between the Bayesian methods and gBLUP. But, superiority between the two methods depends on the specific traits evaluated. For example, in a comparison using dairy cattle data, the accuracy of Bayes B (0.73) was superior to gBLUP (0.64) for milk fat content; however, gBLUP (0.60) was superior to Bayes B (0.55) for milk protein content. In general, Bayes B has the accuracy advantage over gBLUP for traits controlled by smaller numbers of QTNs; otherwise, gBLUP has the advantage (Daetwyler et al. 2010). Nonetheless, given gBLUP’s higher computational efficiency, developing methods within the BLUP framework that have similar or greater prediction accuracy than the Bayes methods for traits with a variety of genetic architectures is highly desirable.

In addition to number of genes, heritability is another important dimension of genetic architecture. Both the Bayes methods and gBLUP are sensitive to heritability and perform poorly for traits with low heritability. Therefore, new methods that are more tolerant to low heritability than Bayes methods and gBLUP are also critically needed.

To address this need, we set out to expand the BLUP alphabet series, targeting the incorporation of two important features: (1) the series retains the computational efficiency of gBLUP and (2) the series adapts to a variety of trait genetic architectures so that prediction accuracy improves. Accordingly, we added two new options within the BLUP framework—one option for traits controlled by a small number of genes and one for traits with low heritability.

## Materials and methods

### Real data

Four published datasets from Arabidopsis, maize, mice, and rice were used in this study. The first three species were used to examine prediction accuracy due to their large amount of available traits. The fourth species (rice) was used to examine computing speed because it has the largest amount of SNPs. Each dataset contained both phenotypes and genetic markers. The mice data came from a heterogeneous stock population kept by The Welcome Trust Centre for Human Genetics (WTCHG) (available at http://gscan.well.ox.ac.uk). This population was generated from crossing eight inbred lines, followed by 50 generations of random mating. The dataset contains 1940 individuals genotyped with 12,227 SNP markers (Neves et al. 2012). The maize dataset contains 282 inbred lines. The genotypes contain 51,182 SNPs (Buckler et al. 2009; Cook et al. 2012). The *Arabidopsis* dataset includes 199 landraces genotyped by 216,130 SNPs and has 81 traits (http://walnut.usc.edu/2010/GWA) for Arabidopsis dataset. The rice data contains 374 inbred lines (Huang et al. 2010). The genotype data contains 842,474 SNPs from genotyping-by-sequencing technology. We found no clear cut between-generations in the *Arabidopsis* and maize datasets.

### Simulated phenotypes

The phenotypes were simulated from real genotypes. SNPs in each dataset were randomly sampled as QTNs to control the additive genetic effects of the simulated phenotypes. The additive genetic effects are the sum of all the QTN effects. The QTN effects followed a standard normal distribution. After the observed additive genetic variance was calculated, residual effects were added to the additive genetic effects to form phenotypes. The residual effects also followed a normal distribution. The variance of the residual distribution was set accordingly so that the proportion of additive genetic effects to the total variance equaled heritability.

### Pedigree-based BLUP

Although we did not use pedigree BLUP in our data analyses, it was the foundation for all the BLUP methods we developed in this study. Individuals’ genetic effects (u) were treated as random effects in a fixed effects and random effects MLM, originally developed for using pedigree to derive kinship among individuals. The model can be expressed as

1 $${\mathrm{y = X\beta + Zu + e}}$$

where y is a vector of phenotypes; β represents unknown fixed effects, including population structure and associated Quantitative Trait Loci (QTLs); and u is a vector of genomic prediction with size *n* (the number of individuals) for unknown random polygenic effects. These random effects follow a distribution with a mean of zero and a covariance matrix of ${G = A\sigma}_a^2{\mathrm{ = }}2K{\mathrm{\sigma }}_a^2$, where *K* = 0.5 A is the pedigree-based kinship with element *K*_ij_ (i, j = 1, 2, …, n) representing the relationship between individuals i and j, *A* = 2 K is additive numerator relationship, and ${\mathrm{\sigma }}_{\mathrm{a}}^2$ is an unknown genetic variance. *X* and *Z* are the incidence matrices for β and u, respectively; **e** is a vector of random residual effects that are normally distributed with a mean of zero and a covariance of ${R = I\sigma}_e^2$, where *I* is the identity matrix and ${\mathrm{\sigma }}_{\mathrm{e}}^2$ is the unknown residual variance.

The full likelihood of observed y, given β, ${\mathrm{\sigma }}_{\mathrm{a}}^2$, and ${\mathrm{\sigma }}_{\mathrm{e}}^2$, is as follows:

2 $${\mathrm{l}}_{\mathrm{F}}\left( {{\mathrm{y}};\,{\mathrm{\beta }},\,{\mathrm{\sigma }}_{\mathrm{a}}^2,\,{\mathrm{\sigma }}_{\mathrm{e}}^2} \right){\mathrm{ = }}\frac{1}{2}\left[ { - {\mathrm{nlog}}\left( {2{\mathrm{\pi \sigma }}_{\mathrm{a}}^2} \right) - {\mathrm{log}}\left| {H} \right| - \frac{1}{{{\mathrm{\sigma }}_{\mathrm{a}}^2}}\left( {{\mathrm{y}} - {\mathrm{X\beta }}} \right)^\prime {H}^2\left( {{\mathrm{y}} - {\mathrm{X\beta }}} \right)} \right]$$

where

3 $${H =}2{K + \delta I}$$

and

4 $${\mathrm{\delta = }}\frac{{{\mathrm{\sigma }}_{\mathrm{e}}^2}}{{{\mathrm{\sigma }}_{\mathrm{a}}^2}}$$

The residual likelihood of observed y, given ${\mathrm{\sigma }}_{\mathrm{a}}^2$ and ${\mathrm{\sigma }}_{\mathrm{e}}^2$, is as follows:

5 $$\begin{array}{*{20}{c}} {{\mathrm{l}}_{\mathrm{R}}\left( {{\mathrm{y}};\,{\mathrm{\sigma }}_{\mathrm{a}}^2,\,{\mathrm{\sigma }}_{\mathrm{e}}^2} \right){\mathrm{ = l}}_{\mathrm{F}}\left( {{\mathrm{y}};\,{\mathrm{\beta }},\,{\mathrm{\sigma }}_{\mathrm{a}}^2,\,{\mathrm{\sigma }}_{\mathrm{e}}^2} \right)} \cr { + \frac{1}{2}\left[ {{\mathrm{qlog}}\left( {2{\mathrm{\pi \sigma }}_{\mathrm{a}}^2} \right) + {\mathrm{log}}\left| {{XX}\prime } \right| - {\mathrm{log}}\left| {{X}\prime {H}^{ - 1}{X}} \right|} \right]} \end{array}$$

where *q* is a rank of *X* and *δ* is solved to maximize (5) by using the EMMA algorithm.

The estimate of β and prediction of u are solved as follows:

6 $$\left[ {\begin{array}{*{20}{c}} \beta \cr u \end{array}} \right] = \left[ {\begin{array}{*{20}{c}} {X\prime X} & {Z\prime X} \cr {X\prime Z} & {Z^\prime Z + \delta A^{ - 1}} \end{array}} \right]^{ - 1}\left[ {\begin{array}{*{20}{c}} {X\prime y} \cr {Z\prime y} \end{array}} \right]$$

### Genomic BLUP

The difference between gBLUP and pedigree BLUP is that gBLUP substitutes the pedigree-based kinship with the marker-based kinship. A variety of algorithms can be used to derive kinship from markers. Comparisons among these algorithms were conducted for both GWAS and genomic prediction. In general, these algorithms have the same statistical power for GWAS and similar accuracy for genomic prediction (Endelman 2011; Forni et al. 2011). We used the efficient algorithm by VanRaden, as follows:

7 $$2{K =}\frac{{{WW}^\prime }}{{2{\sum} {{\mathrm{p}}_{\mathrm{i}}\left( {1 - {\mathrm{p}}_{\mathrm{i}}} \right)} }}$$

where W is a centralized genotype matrix with rows as individuals and columns as markers, and _pi_ is the frequency of the second allele for the ith marker (VanRaden 2008).

### SUPER BLUP

Inspired by taBLUP that uses weighted markers (Smith and Gibson 1997; Zhang et al. 2010a, 2010b; Su et al. 2014; Zhang et al. 2016), we derived kinship from estimated QTNs. Ideally, for a specific trait, the kinship among individuals should be defined by the genes that control the trait. Including additional markers will dilute the precision of kinship among individuals for the specific trait. We tried to solve two major problems that limit the prediction accuracy of taBLUP. The first problem was the duplicate use of associated markers in linkage disequilibrium (LD). These markers appeared on the same peaks in the GWAS results and had similar weights. The second problem was that the majority of markers were not associated with particular traits. Although individually these markers are assigned a small weight, together they can contribute significantly to kinship. Therefore, these markers needed to be identified and eliminated.

**Fig. 1:**
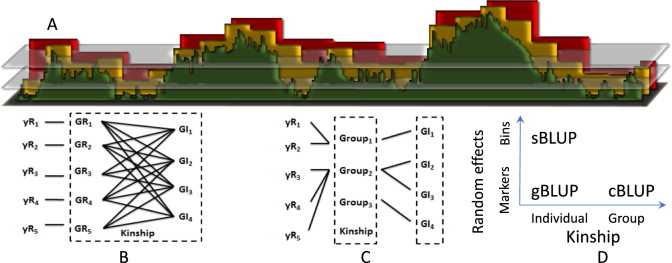
Development of the BLUP alphabet. The best linear unbiased prediction (BLUP) of random effects changes according to the base of the random effects or the variance structure of the random effects. The initial base of the random effects is individuals. The variance structure of individuals is defined by their kinship multiplied by the genetic variance among individuals. Kinship can be derived from markers or selected bins (**a**). Each marker is a bin when the bin size is a single base pair. The selection of markers is based on the strength of their associations with a trait of interest, e.g., negative log P values are indicated by the green peaks. As bin size increases, indicated by the yellow and red blocks, multiple markers belong to the same bin. The strength of a bin is represented by the marker with strongest association within the bin. The selection of bins is determined by the threshold of association, indicated by the horizontal layers. The bin size and number of bins selected are optimized by the maximum likelihood method, introduced by the SUPER (Settlement of MLM under progressively exclusive relationship) algorithm. Either markers or bins can be used to derive kinship to define genetic (g) similarity among individuals (**b**). The kinship includes two types of individuals, those with phenotypes (**y**), and those without phenotypes. Individuals with phenotypes are classified as Reference (**r**), otherwise as Inference (**i**). Both Reference individuals and Inference individuals are clustered into groups using a compressed mixed linear model (**c**). When the groups are clustered, kinship among individuals is replaced by the kinship among groups accordingly. Consequently, the original genomic BLUP (gBLUP) has evolved into SUPER BLUP (sBLUP) by shifting markers to bins, or into compression BLUP (cBLUP) by shifting individuals to groups (**d**)

Both pedigree-based BLUP and marker-based genomic BLUP use the average kinship across the genome. That is, kinship is not specific to a particular trait. However, studies have demonstrated that using trait-specific kinship results in higher prediction accuracy (Zhang et al. 2010a, 2010b) and higher statistical power (Wang et al. 2014). In these studies, the trait-specific kinship was derived from either the associated markers or by weighting all markers. Here, we derived kinship by selecting the associated markers using a likelihood method, and excluding the remaining markers. This method was developed in our previous study on GWAS and named Settlement Under Progressively Exclusive Relationship (SUPER). In SUPER, the real QTNs are estimated as bins, with bin size and number optimized using a likelihood method (Fig. 1a). These estimated QTNs are named pseudo QTNs. We used the pseudo QTNs to derive kinship among individuals. For the convenience of illustration, we named BLUP based on the SUPER method SUPER BLUP (sBLUP). One slight difference between deriving kinship for sBLUP and deriving kinship for GWAS is that sBLUP does not have exclusion process, which is sued to exclude the associated markers that are in LD with the testing marker for GWAS. All markers that maximize the likelihood for a particular trait are used to derive kinship for sBLUP.

The difference between gBLUP and sBLUP is that gBLUP uses all the markers and sBLUP uses the associated markers to derive kinship in formula (7). In sBLUP, all associated markers are grouped into bins based on their locations on chromosomes (Hu et al. 2012). After GWAS is performed on all markers, the entire genome is divided into bins with size of s base pairs. Each bin is represented by the most significant marker within the bin. The top *t* significant bins are selected as a *W* matrix for calculating kinship using formula (7). The size of bins (*s*) and number of bins selected (*t*) are optimized to maximize the following likelihood, which has the same format as formula (5):

8 $${\mathrm{l}}_{\mathrm{R}}\left( {{\mathrm{y}};\,{K},\,{\mathrm{\sigma }}_{\mathrm{a}}^2,\,{\mathrm{\sigma }}_{\mathrm{e}}^2} \right) = {\mathrm{l}}_{\mathrm{R}}\left( {{\mathrm{y}};\,{W},\,{\mathrm{\sigma }}_{\mathrm{a}}^2,\,{\mathrm{\sigma }}_{\mathrm{e}}^2} \right) = {\mathrm{l}}_{\mathrm{R}}\left( {{\mathrm{y}};\,{s},\,{t},\,{\mathrm{\sigma }}_{\mathrm{a}}^2,\,{\mathrm{\sigma }}_{\mathrm{e}}^2} \right)$$

The conventional gBLUP uses the kinship in formula (3), derived from using all the markers as *W*; whereas, sBLUP uses the kinship derived from the selected bins through formula (8) as *W*.

### Compressed BLUP

In a previous study (Zhang et al. 2010a, 2010b), we replaced random effects from individuals by random effects from their corresponding groups. Individuals were compressed (clustered) into groups based on kinship among individuals. We used this method for GWAS and demonstrated that it improves statistical power for mapping genes. For genomic prediction in this current study, we first clustered individuals into groups, whether or not these individuals had phenotypes. Then, we determined the optimum grouping that gave the best likelihood (Fig. 1b–d) based on individuals with phenotypes. We named this new BLUP variation compressed BLUP, abbreviated as cBLUP. The conventional gBLUP is an extreme case of cBLUP, where each individual is its own group. Theoretically, cBLUP should perform better than, or at least as well as, gBLUP, because cBLUP choose the grouping with the maximum likelihood.

With individuals in gBLUP replaced by the corresponding groups in cBLUP, the kinship among individuals is also replaced by the kinship among groups. The kinship between two groups is defined as the average kinship between the individuals of the two groups. The kinship of a group itself is the average kinship among individuals in the group. For a specific clustering method, the grouping and the kinship among the groups are completely defined by the number of groups. Therefore, likelihood under the compressed MLM can be defined as follows:

9 $${\mathrm{l}}_{\mathrm{R}}\left( {{\mathrm{y}};\,{\mathrm{grouping}},\,{\mathrm{\sigma }}_{\mathrm{a}}^2,\,{\mathrm{\sigma }}_{\mathrm{e}}^2} \right){\mathrm{ = l}}_{\mathrm{R}}\left( {{\mathrm{y}};\,{g},\,{\mathrm{\sigma }}_{\mathrm{a}}^2,\,{\mathrm{\sigma }}_{\mathrm{e}}^2} \right)$$

where *g* is the number of groups.

For *n* individuals and a given clustering algorithm, the clustering tree is determined only by the kinship among individuals, with *n* possible grouping levels, *g* = *n*, *n*−1, …, 2, and 1. To reduce computing time, an option is to examine the likelihood of n possibilities with intervals. For each grouping level, the likelihood is determined by the individuals with phenotypes. For *p* individuals with phenotypes, the dimensions of *y*, *u*, and *Z* matrices in (1) are *p* by 1, g by 1, and *p* by *g*, respectively. Consequently, the kinship among groups has dimensions of *g* by *g*. Some groups may contain both types of individuals, with and without phenotypes. Some groups may contain only the individuals without phenotypes. Even in the latter case, the prediction formula is still the same as (6). The only difference is that kinship among individuals is replaced by kinship among groups.

### Bayesian LASSO

With different settings of priors, the Bayesian alphabet series offers a variety of methods for performing GS. We chose Bayesian LASSO (Bayesian Least Absolute Shrinkage and Selection Operator) because, in addition to the criteria of computing speed and software availability, it has least assumptions among the Bayesian methods. The fundamental assumption is that the effect of a marker has its own distribution characterized by the variance of the genetic effect. The variance follows a double exponential distribution without further hyper-parameters. The other consideration in choosing Bayesian LASSO was that the differences between Bayesian LASSO and other Bayesian methods are well-documented (Moser et al. 2009; Heslot et al. 2012; Neves et al. 2012).

Bayesian LASSO was implemented in a commonly used R package, “BLR” (Pérez et al. 2010; Heslot et al. 2012). This package runs a Gibbs sampler on a Bayesian regression model with multiple optional effects. These effects include: (1) fixed effects with a flat prior, (2) RR with random effects following an identical independent distribution, (3) correlated random effects with variance structure defined by a kinship matrix, and (4) the random effects of Bayesian LASSO. In this study, we fit only the fixed effects and the random effects of Bayesian LASSO. The fixed effects included the first three principal components for all species. In this study, we set 80,000 iterations and 60,000 as the burning part for all Bayesian LASSO experiments.

### Cross-validation

For each dataset, individuals were randomly sampled into five groups. One group was treated as the inference and the other four groups were treated as the reference. The phenotypes of the reference panel were used to derive the genomic prediction (Weber et al. 2012). The phenotypes of the inference panel were masked and only the genotypes of the inference were used to derive the genomic prediction. GWAS and sBLUP were performed on the individuals of the reference only. The Pearson correlation coefficient was calculated between predicted and observed phenotypes in the inference group (Crossa et al. 2010; Ober et al. 2012). The inference group was rotated to another group until every group was treated as the inference. The mean of the five correlation coefficients was used as the prediction accuracy (Zhou et al. 2016). The random grouping and prediction accuracy calculation process was repeated 40 times for all BLUP methods and 20 times for Bayesian LASSO. We reported the means and standard errors of the replicates.

## Results

To show how these two new BLUP variations work and their advantages over both gBLUP and Bayesian LASSO, we conducted a series of studies on both simulated phenotypes and real phenotypes in three species (Arabidopsis, mice, and maize). The number of individuals and markers in each dataset are summarized in Table S1. Prediction accuracy was examined by a five-fold cross-validation. The predicted phenotypes of inference were then compared to their true phenotypes to evaluate prediction accuracy.

### Kinship based on true or estimated QTNs

For a specific trait, the kinship among individuals is best defined from only the true QTNs. With simulated traits, we compared the prediction accuracies that resulted from using four different sets of markers to define kinship: (1) true QTNs, (2) all available markers, (3) true QTNs plus 1000 Non-QTN Markers (NQMs) for dilution, and (4) estimated QTNs by using the SUPER algorithm under the assumption that their locations are unknown. The set of true QTNs mimics an ideal situation, which is unrealistic in practice. The set of all available markers corresponds to the kinship-derivation method in conventional gBLUP. The set of true QTNs plus NQMs demonstrates the impact of dilution as a consequence of including NQMs. The set of estimated QTNs corresponds to the method used in sBLUP.

The simulated traits were controlled by 10 QTNs at three levels of heritability (0.25, 0.5, and 0.75). The QTNs were randomly sampled from a total of 12,227 available markers on 1940 mice individuals from The Welcome Trust Centre for Human Genetics (WTCHG) dataset (http://mus.well.ox.ac.uk/mouse/HS/). Model fit and prediction accuracy were evaluated through five-fold cross-validation. Model fit was evaluated as twice the negative log likelihood (−2LL) in the reference population. As both cBLUP and sBLUP do not change the number of fixed effects, other criteria, such as Akaike Information Criterion (AIC) or Bayesian Information Criterion (BIC), stay the same trend as likelihood. The lower the −2LL value, the better the model fit. Prediction accuracy was evaluated as the Pearson correlation coefficient between the observed and predicted phenotypes in the inference population. The phenotypes of inferences were not used for estimating QTNs to ensure independent tests on inferences.

**Fig. 2:**
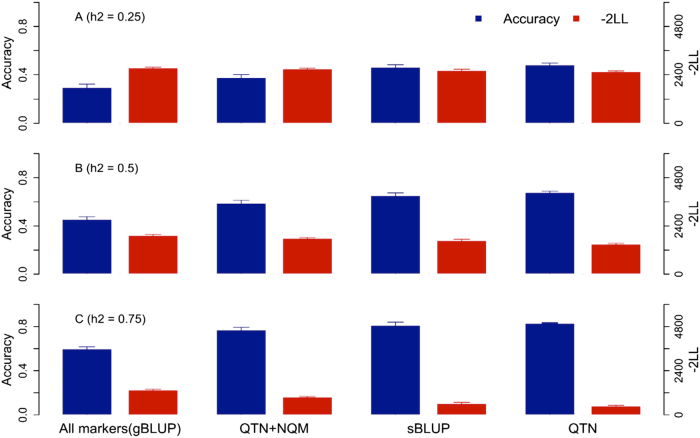
Impact of kinship derived from different types of markers on prediction accuracy and model fit. Prediction accuracy and model fit were evaluated on simulated mouse traits controlled by 10 QTNs at three levels of heritability. The heritability (h2) was set to 0.25, 0.5, and 0.75 (**a**–**c**, respectively). QTNs were randomly sampled from a total of 12,227 available markers on 1940 mice individuals from the WTCHG dataset. Four types of markers were used to derive kinship: (1) all markers (method of gBLUP), (2) 10 true QTNs plus 1000 randomly selected Non-QTN Markers (NQM), (3) estimated QTNs from SUPER (method of sBLUP), and (4) 10 true QTNs. We compared the impact of the different kinship derivations on prediction accuracy as the Pearson correlation coefficient between predicted and observed phenotypes, and on model fit as twice the negative log likelihood (−2LL). The accuracy (blue bars) and −2LL (red bars) are displayed as the means of 40 replicates. The standard errors are indicated by the whiskers on the bars. Each replicate used a five-fold cross-validation. Inferences were used to evaluate prediction accuracy and references were used to evaluate −2LL. The phenotypes of inferences were not used for estimating QTNs in sBLUP to ensure independent tests on inferences. As expected, the set of estimated QTNs (i.e., sBLUP) performed a bit worse than the ideal scenario (true QTNs). However, sBLUP performed better than QTN + NQM and much better than all markers (gBLUP)

The means and standard errors for the 40 replicates are illustrated in Fig. 2. Whether model fit in reference or prediction accuracy in inference, the set of true QTNs performed the best across the different levels of heritability. The set of all the available markers (i.e., gBLUP) performed the worst. Adding 1000 NQM to the true QTNs reduced model fit compared to the set of true QTNs. Although the set of estimated QTNs (i.e., sBLUP) performed worse than the set of true QTNs, it performed better than the set of true QTNs plus NQM, and much better than gBLUP.

### Substitution of individuals with groups

**Fig. 3:**
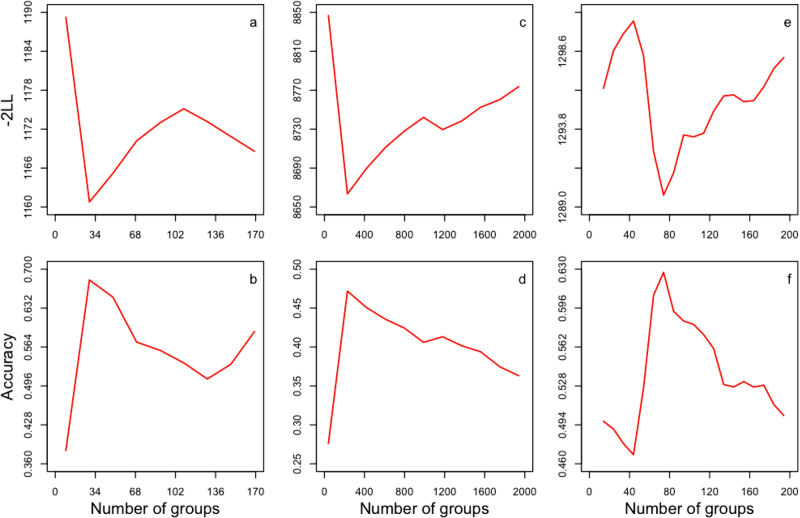
Impact of clustering individuals to groups on model fit and prediction accuracy. The impact was evaluated for three real traits in three species: short-day vernalization (SDV), weight growth intercept, and plant height for Arabidopsis, mice, and maize, respectively. The entire population in each species was randomly divided into reference (80%) to exam model fit and the rest as inference to exam prediction accuracy. Clustering individuals into groups was based on kinship among individuals from both references and inferences; however, the phenotypes of inferences were masked. When number of groups equaled number of individuals, the prediction was equivalent to the prediction resulting from the conventional genomic BLUP (gBLUP) method. When a group contained more than one individual, the prediction for a group was used as the prediction for each individual within the group. The phenotypes of references were used to evaluate the model fit (top panel: **a**–**c**), as indicated by twice the negative log likelihood (−2LL). Prediction accuracies for cBLUP were evaluated as the correlations between predicted and observed phenotypes of inferences (bottom panel: **d**–**f**). Initially, both model fit and prediction accuracy increased with increasing number of groups, but decreased after reaching an optimum peak. The optimum peak of model fit corresponded to the optimum peak of prediction accuracy for compressed BLUP (cBLUP). This trend was consistent for all the traits in the three species we examined, as demonstrated on a replicate for Arabidopsis (**a**, **d**), mice (**b**, **e**), and maize (**c**, **f**). The trend was also consistent across replicates of randomly assigning reference and inference, however, the magnitude of likelihood, accuracy, and number of groups at the peaks varied slightly among replicates

To evaluate the impact of substituting individuals with their corresponding groups (i.e., cBLUP method), we examined model fit and prediction accuracy on real traits in three species: Arabidopsis, mice, and maize (Fig. 3). For the real traits, we used short-day vernalization (SDV), weight growth intercept, and plant height for Arabidopsis, mice, and maize, respectively. These traits had the most number of records in each species. All available markers were used to derive kinship among individuals.

We clustered individuals into groups based on kinship among individuals from both references and inferences; however, the phenotypes of inferences were masked. That is, only the phenotypes of references were used to evaluate the model fit, based on −2LL. Prediction accuracies were calculated as the correlations between predicted and observed phenotypes in the inference populations. The prediction for a group was used as the prediction for each individual within the group. We varied the number of groups from one to the total number of individuals. The average number of individuals per group was defined as the compression level in a previous study (Zhang et al. 2010a, 2010b). The minimum level of compression occurs when each individual is its own group. When number of groups equaled number of individuals (i.e., the far right side of each plot in Fig. 3), the prediction was equivalent to that of the conventional gBLUP. The maximum level of compression occurs when all individuals belong to one group. In this case, the BLUP of the group is confounded with the overall mean and is therefore irrelevant.

Both model fit and prediction accuracy initially increased with increasing numbers of groups, but then each decreased after reaching an optimum peak (Fig. 3**)**. This trend was the same as that of statistical power in GWAS in response to varying the compression level (Zhang et al. 2010a, 2010b). The optimum compression level varied across species, subpopulations, and traits. We also found that the model fit in the reference population and the prediction accuracy in the inference population changed correspondingly. The compression level (or the number of groups) with the best model fit in the reference population corresponded to the optimum compression level with the best prediction accuracy in the inference population. These results show that, in practice, model fit is a useful criterion for deciding which compression level to use for the final (optimum) genomic prediction in cBLUP. Therefore, we used the prediction accuracies at the optimum peak of model fit as the final prediction accuracies for cBLUP. The accuracies for cBLUP were higher than gBLUP in all three species.

### Performance on simulated traits with different genetic architectures

To fully understand which BLUP or Bayes method should be used with different genetic architectures, we examined prediction accuracies on simulated traits. Genetic architecture is defined in two dimensions. One dimension is complexity, based on number of QTNs. Four levels of QTN numbers were evaluated: 5, 100, 500, and 1000. The QTNs were sampled from 12,227 real markers genotyped on 1940 mice individuals from the WTCHG dataset. The second dimension is heritability. Four levels of heritability were evaluated: 10, 25, 50, and 75%. We examined three BLUP methods (gBLUP, sBLUP, and cBLUP) and Bayesian LASSO with five-fold cross-validation.

**Fig. 4:**
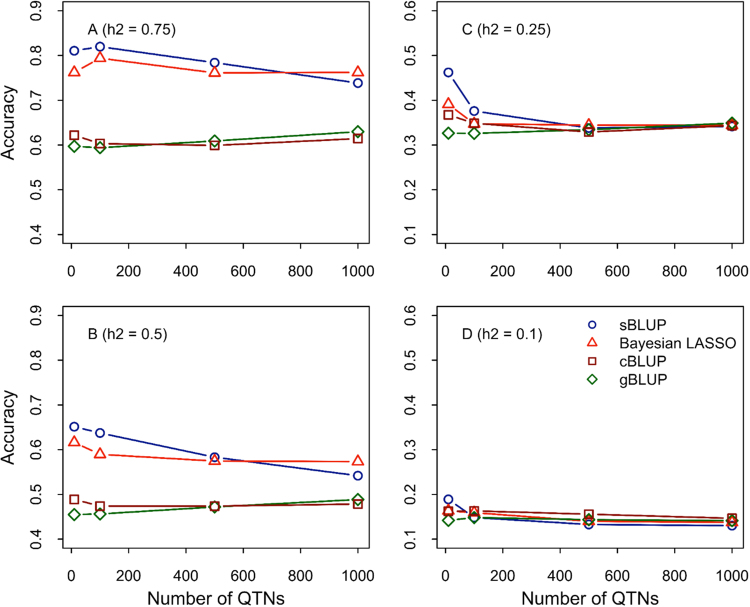
Interaction between prediction methods and genetic architecture. Prediction accuracies were evaluated on mouse phenotypes simulated with different numbers of Quantitative Trait Nucleotides (QTNs) at different heritabilities. The QTNs were sampled from 12,227 markers genotyped on 1940 mice individuals from the WTCHG dataset. The heritabilities (h^2^) were set to 0.75, 0.5, 0.25, and 0.1 (**a**–**d**, respectively). Five-fold cross-validation was conducted to evaluate prediction accuracies by comparing four methods: sBLUP, Bayesian LASSO, cBLUP, and gBLUP. The cross-validations were replicated 40 times when gBLUP, cBLUP, and sBLUP were used and 20 times when Bayesian LASSO was used. The first 80,000 iterations were used as “burn in” and the next 60,000 iterations were used to derive Bayesian estimations. The advantage of the new developed method are under condition of less QTNs (sBLUP) and low heritability (cBLUP)

For a trait controlled by a few QTNs with high heritability, we found that the prediction accuracy of sBLUP is superior to any other method (Fig. 4a, b). For traits with low heritability (Fig. 4d), cBLUP is superior to any other method except in situations where traits are controlled by a few QTNs. In all cases, cBLUP is either similar or superior to gBLUP (Fig. 4a–d). For traits with high heritability, Bayesian LASSO is superior to any other method except in situations where traits are controlled by a small number of QTNs (Fig. 4a, b).

Although ranking the genetic merit of individuals is the primary objective of genomic prediction, understanding the magnitude of predictions compared with their true values is also of academic interest. The phenomenon known as inflation of prediction, where BLUPs shrink as they approach zero, is well-documented. Thus, we were interested in exploring how the different BLUP methods performed relative to inflation under the different genetic architectures. We simulated a trait controlled by 5, 100, 500, and 1000 QTNs under different heritabilities (0.1, 0.25, 0.5, and 0.75).

As expected, the regression coefficients of predicted breeding values over the true breeding values were less than one for all methods under all circumstances due to shrinkage of BLUPs. However, their magnitude of inflation were different. sBLUP demonstrated the least inflation when the number of QTNs was small (5 and 100), but the most inflation when the number was large (500 and 1000). This result suggested again that sBLUP performs best when traits are controlled by a small number of QTNs. For a trait controlled by a larger number of QTNs (500 and 1000), the advantage of gBLUP and cBLUP over sBLUP strengthened with increased heritability. In all circumstances, cBLUP outperformed gBLUP. This trend for inflation followed the same trend for prediction accuracies (Figure S1).

### Performance on real traits across species

**Fig. 5:**
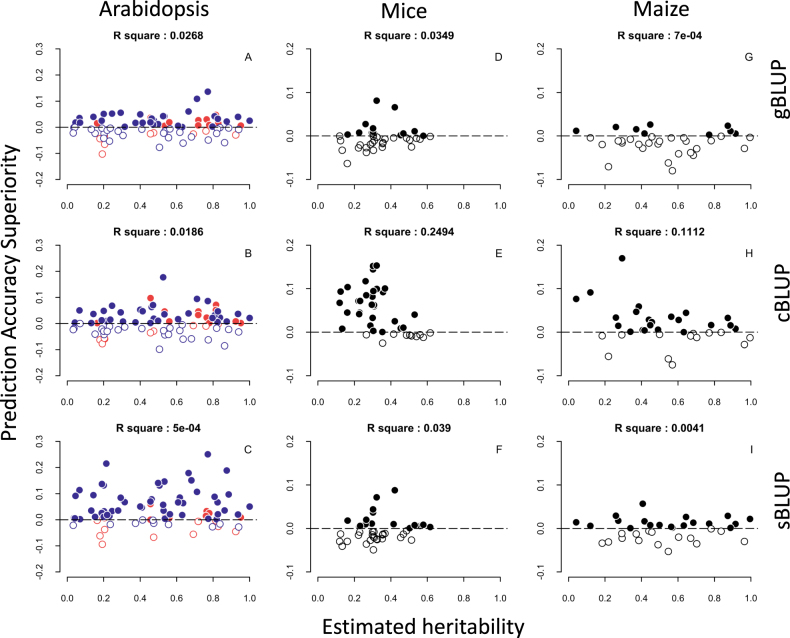
Superiority of BLUP methods over Bayesian LASSO on real traits. Three BLUP methods (gBLUP, cBLUP, and sBLUP) were compared with Bayesian LASSO. Superiority was defined and calculated as the difference in prediction accuracy between each BLUP method and Bayesian LASSO. Each trait is presented as a dot and positioned according to its heritability and superiority value for the three BLUP methods. A dot is filled solid if a BLUP method is superior to Bayesian LASSO; otherwise, a dot is outlined. We evaluated 81, 41, and 35 real traits from Arabidopsis (**a**–**c**), mice (**d**–**f**) and maize (**g**–**i**), respectively. The Arabidopsis data contained 21 traits on flowering time that were classified as complex traits in a previous study (cite). The complex traits from Arabidopsis are colored red; the other traits (simple) are colored blue (**a**–**c**). We did not differentiate complexity for the traits in maize or mice. All traits are colored black in these two species (**d**–**i**). For most of the simple traits from Arabidopsis (blue dots), sBLUP is superior to Bayesian LASSO (**c**). For the mice traits with low heritability (<40%), cBLUP is superior to Bayesian LASSO (**e**). In maize, Bayesian LASSO is superior to gBLUP for most of the traits, but not compared with cBLUP or sBLUP(**g**)

We examined the three BLUP methods (gBLUP, cBLUP, and sBLUP) and Bayesian LASSO on a total of 157 real traits from three species: Arabidopsis (81), mice (41), and maize (35). The heritability of each trait was estimated as the proportion of estimated genetic variance among individuals to total variance. All traits are presented in Fig. 5 as the heritability and superiority of each BLUP method over the Bayesian LASSO method.

The superiority of each method depends on both the complexity and heritability of the trait being analyzed. Among the 81 traits in Arabidopsis, 21 are complex traits associated with flowering time. The other 60 traits are relatively simple, relating to development and defense. For the 60 simple traits, we observed that sBLUP performed much better than Bayesian LASSO (Fig. 5 and S2). The heritabilities of traits in mice are much lower than the heritability of traits in Arabidopsis and maize. The 41 traits in mice had a mean estimated heritability of 30, versus 55% in maize and 50% in Arabidopsis. For mice traits with low heritability, cBLUP performed much better than Bayesian LASSO. Out of the total 157 traits, Bayesian LASSO had an advantage over sBLUP and cBLUP for only 21 traits (13%) (Figure S4). These observations on real traits reflected the same trend we observed on simulated traits.

Among the three species (Arabidopsis, maize, and mice), mice had a clear family structure defined by the female parents. The 1940 individuals were from 160 female parents. This structure allowed us to create different levels of relatedness between reference and inference, which helped to investigate the relationship between prediction accuracy and level of relatedness. We chose the trait with the most phenotype records, weight growth intercept, to investigate the impact of relatedness on prediction accuracy through five-fold cross-validation (Figure S3).

Under the scheme of family selection with one individual from each family, the relatedness between reference and inference was defined as the average relatedness among families. No individuals in the inference had family members in the reference. As expected, both gBLUP and cBLUP had low prediction accuracies, and we found no significant difference between the two methods. When we increased the number of individuals per family to five, for a total of 800, the chance that individuals in the inference had family members in the reference greatly increased. Consequently, prediction accuracy improved for both cBLUP and gBLUP, with cBLUP’s improvement much higher than gBLUP’s. cBLUP’s advantage over gBLUP remained when individuals were sampled with an average of 5 individuals per family; that is, the number of individuals per family varied. However, when the average number of individuals per family was reduce to one, the prediction accuracies decreased and the advantage of cBLUP over gBLUP disappeared. As expected, the prediction accuracies that resulted from sampling exactly one individual per family were lower than sampling an average of one individual per family. In the latter case, because the selection of individuals was random, any one individual in the inference could have had family members in the reference.

### Superiority domains of different methods

**Fig. 6:**
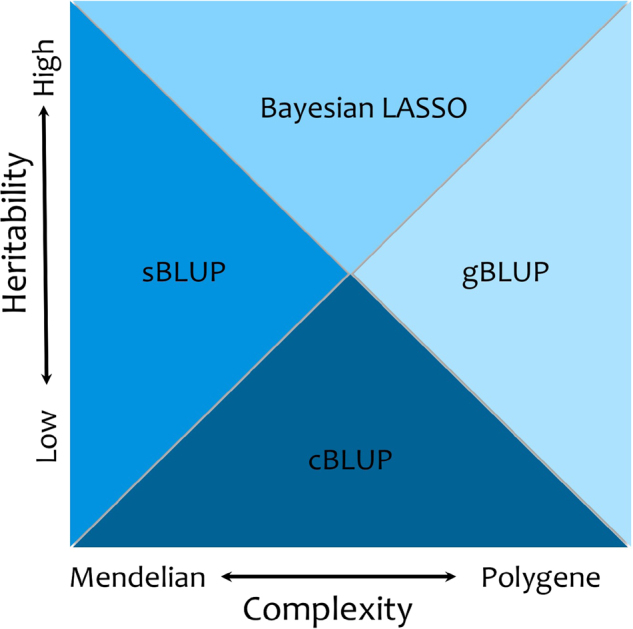
Working domains of prediction methods corresponding to genetic architecture. Genetic architecture is defined by two dimensions. One dimension is complexity, defined by the number of genes that control a trait. The simplest traits are Mendelian traits, which are controlled by only a few genes. The complex traits are controlled by many genes. The other dimension is heritability. Each prediction method has its own dominant domain of genetic architecture defined by different levels of the two dimensions. These domains are conceptually illustrated by the areas colored with different shades of blue. For example, for a trait controlled by a small number of genes with high heritability, sBLUP is superior to others; whereas, for a trait with low heritability, cBLUP is much better than the other methods

Results of our comparisons on simulated and real traits from the three species suggest that each of the BLUP methods and Bayesian LASSO has its own domain of superiority according to genetic architecture (Fig. 6). These domains are defined by different levels of the two dimensions of genetic architecture: heritability and complexity. Complexity is determined by the number of genes that control a trait. The simplest traits (i.e., lowest complexity) are Mendelian traits, which are controlled by only a few genes. The complex traits are controlled by many genes.

The sBLUP method is superior for traits with fewer genes. Bayesian LASSO performs well for traits with high heritability. The gBLUP method is best used with complex traits controlled by many genes. In most cases, cBLUP is superior to gBLUP, especially for traits with low heritability. With increased heritability and number of genes, the superiority of cBLUP over gBLUP disappears.

### Computing time

The complexity of computing time for the BLUP methods is *O*(*mn*^*2*^ + *n*^*3*^) with P3D (population parameter previously determined) algorithm (Zhang et al. 2010a, 2010b), where *n* is the number of individuals and *m* is the number of markers. The BLUP methods use a two-step calculation process. The first step calculates kinship. The second step solves the MLM equations. The time complexity of the kinship calculation is *O*(*mn*^*2*^). The time complexity of the MLM step is O(*n*^*3*^), required to produce the inverse of the kinship matrix. Although cBLUP and sBLUP increase the number of inversions, the complexity remains the same relative to *n*. Because the number of markers is irrelevant when solving MLM, cBLUP has the same level of computing time complexity as gBLUP, regardless of number of markers. During the optimization of grouping, computing time is reduced when more individuals are clustered into the same groups, compared to the initial stage, i.e., gBLUP.

**Fig. 7:**
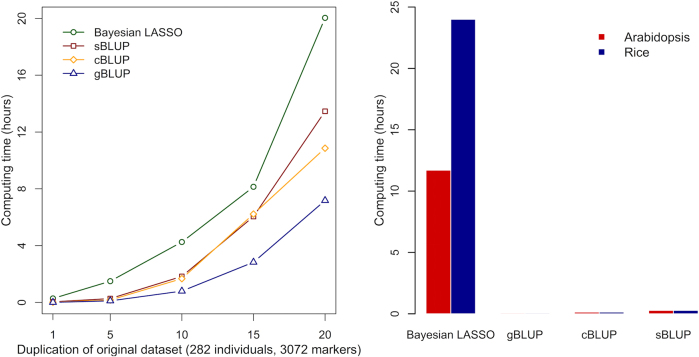
Computing times of genomic prediction methods. The computing times were examined on both synthetic and real datasets. The synthetic datasets were created from an original dataset of maize with 282 individuals and 3082 markers. The original dataset was duplicated 5, 10, 15, and 20 times for both individuals and SNPs (**a**). The largest synthetic dataset (duplicated 20 times) contained 5640 lines and 61,640 SNPs. The real datasets contained 199 individuals genotyped with 216,131 SNPs in Arabidopsis and 374 individuals genotyped with 842,474 SNPs in rice, respectively (**b**). Both cBLUP and sBLUP have similar computing efficiency as gBLUP, which is much more efficient than Bayesian LASSO for situations with more markers than individuals

The computing time complexity of Bayesian LASSO is *O*(*mnl*), where *l* is the length of the Markcov Chain (MC). For a given length of MC, the complexity of Bayesian LASSO is linear to both number of individuals and markers. However, the actual computing time is much greater than the BLUP methods with large numbers of markers because the MC Monto Caro (MCMC) length is long and also related to both *m* and *n*. Thus, with more markers, Bayes methods are slower than BLUP methods. In our datasets, the number of markers was much larger than the number of individuals. For example, ratios of markers to individuals (*m*:*n*) in Arabidopsis and rice reached 1086:1 and 2252:1, respectively. In such cases, BLUP methods are superior to Bayes methods. The observed computing times were 14 and 25 h with Bayesian LASSO for Arabidopsis and rice, respectively. In contrast, all the BLUP methods completed these analyses within 30 min (Fig. 7b).

Even for a dataset with an *m*:*n* of 10, the computing time of Bayesian LASSO increased much faster with increasing data size (determined by both markers and individuals) compared to BLUP methods. We compared the computing times of the four methods (Bayesian LASSO, gBLUP, cBLUP, and sBLUP) on multiple synthetic datasets that were created from an original maize dataset with 282 individuals and 3082 markers. The original dataset was duplicated 5, 10, 15, and 20 times for both individuals and SNPs. The largest dataset (duplicated 20 times) contained 5640 lines and 61,640 SNPs. Bayesian LASSO was the most time-consuming. The most efficient method was gBLUP. Due to the additional computation necessary for clustering and selecting estimated QTNs, cBLUP, and sBLUP are less computationally efficient than gBLUP (Fig. 7a).

## Discussion

Kinship is a major element of MLM. Ideally, kinship should precisely define the variance structure of random individuals’ genetic effects for a trait of interest. When another trait is evaluated, the variance structure should change. However, adapting to this change is impossible for the pedigree-based BLUP (pBLUP) method, in which kinship is based on pedigree. Pedigree-based kinship remains constant and represents the average kinship across traits. Pedigree-based kinship not only lacks the ability to adjust for different traits of interest, but also lacks the ability to differentiate among full sibs. Even when variation exists among full sibs, pedigree-based kinship does not distinguish pairwise kinship among them. In contrast, these magnificent variations became evident when we used genetic marker-based kinship methods. In this study, we not only changed the markers used to define kinship among individuals, but also changed the kinship among individuals into kinship among groups. These changes created extra flexibility for BLUP and, in turn, the opportunity to expand the BLUP alphabet series, adaptable to the different genetic architectures of complex traits.

### Realized kinship

The kinship among some individuals, such as full sibs, are exactly the same based on pedigree. However, their kinship is highly likely to be different based on genetic markers. One pair of full sibs may share more common markers than another pair of full sibs due to multiple reasons, for example, Mendelian sampling. Studies have shown substantial variations in kinship among full sibs (Lukić et al. 2015; Vela-Avitúa et al. 2015). Taking a bi-parental population as an example, the proportion of alleles from a specific parent can range from 20 to 80% among full sibs. By using pedigree-based kinship, these differences are masked because kinship is the same among all full-sib pairs. However, marker-based kinship methods reveal the actual similarity among individuals and, thus, partially explain why gBLUP is more accurate than pBLUP (Hayes et al. 2009b; Habier et al. 2010).

### QTN-based kinship

The chance of finding associated markers is higher for traits controlled by a few genes compared to traits controlled by many genes. Consequently, sBLUP is the favored genomic prediction method for traits controlled by a small number of genes because sBLUP uses a select number of estimated (pseudo) QTNs to derive kinship. sBLUP also has the advantage of being able to fully use dense markers such as sequence data. In such cases, chances are greater for finding markers in strong LD with genes controlling the trait of interest. In contrast, genomic predictions with gBLUP is based on kinship derived from all markers. Because kinship calculated from all genetic markers remains stable after the number of genetic markers reaches a certain level, prediction accuracy with gBLUP does not increase with increased marker density. Consequently, dense genetic markers (e.g., sequencing data) provide more information than gBLUP can effectively use. In contrast, the use of dense markers enhances the prediction accuracy of sBLUP and the Bayesian methods.

### Group-based kinship

All of the above derived kinships—pBLUP, gBLUP, and sBLUP—define the genetic relatedness among individuals. Consequently, individuals are fit as random effects. Alternatively, for each of these kinships, individuals can be clustered (compressed) into groups and groups can then be fit as random effects instead of individuals. Therefore, the cBLUP procedure can be applied to any type of kinship among individuals. In this study, we applied cBLUP to all the kinships except the pedigree kinship. When we applied compression to kinship from sBLUP to create a combination, compressed SUPER BLUP (csBLUP), the compression failed to boost accuracy over sBLUP. This result makes sense because kinship in sBLUP is defined by a small number of estimated QTNs. That is, kinship has a much smaller number of different kinship elements, which is similar to kinship among groups.

cBLUP works to boost accuracy when kinship is derived from all markers for two reasons. First, similar to why compression works for GWAS (Zhang et al. 2010a, 2010b; Li et al. 2014), individuals in the same group are more related than individuals from different groups. When individuals are closely related, their phenotypic variations are most likely from error. Each individual can be considered a replicate that represents the common genetic background of individuals in the group. The group genetic effects are better represented by the multiple measurements through individuals.

Second, with cBLUP, individuals are predicted based on their group effects. Thus, the likelihood that an individual of inference will belong to a group containing individuals of reference (with phenotypes) is high. The individuals will share the same prediction. On other hand, the chance is small that an individual of inference will belong to a group in which no individuals have phenotypes. The prediction of the group effect is calculated based on other groups with individuals having phenotypes. This situation is true for every individual of inference by using regular gBLUP. Each individual must be predicted based on other individuals in reference.

One could argue that the genetic differences among individuals in the same group might diminish under the compressed MLM. However, individuals are clustered into the same group for two reasons. First, these individuals are closely related; only minor genetic differences exist among them. Second, most of the phenotypic differences among individuals in the same group are caused by residual errors. In this case, individual-based BLUP, such as gBLUP, will result in different genomic predictions among individuals in the same group; but, these differences will be more likely due to error.

### Infrastructure of kinship methods

The pBLUP method, based on pedigree-based kinship, has been successfully used for genetic evaluation in animals and plants for many decades. Many pBLUP software packages have been developed in both public and private sectors. Compared to the Bayesian methods, gBLUP, sBLUP, and cBLUP have the advantage of easily integrating into the existing genetic evaluation infrastructure that uses pedigree to derive kinship. The only modification required for gBLUP is the replacement of kinship based on pedigree by kinship based on genetic markers. Other minor modifications may also be required for the different types of marker-based kinship derivation used in the other BLUP alphabet methods. For example, the implementation of cBLUP is similar to gBLUP, but additional algorithms will be needed for the individual clustering analyses, group kinship calculations, and optimum grouping assignments.

### Computing time

Genomic prediction with the BLUP alphabet series may involve GWAS, estimation of QTNs, and clustering individuals into groups. Yet, computing time complexity remains the same as conventional gBLUP in terms of number of individuals and markers. For the BLUP methods, computing time complexity is linear to number of markers. Currently, the number of markers is larger than the number of individuals; thus, BLUP methods have the advantage over the Bayesian methods. With the computer software package, Genome Association and Prediction Integrated Tool (GAPIT) (Tang et al. 2016), we analyzed a large dataset, containing over 10,000 individuals and 250,000 SNPs, on a 16G-memory computer. cBLUP finished both GWAS and genomic prediction in just three days. Based on a test with a smaller dataset, we projected that the Bayesian LASSO method would take several years to analyze this same large dataset. However, the trend may change in response to decreasing sequencing costs. By the time the number of individuals becomes greater than the number of markers, BLUP methods will need to be adjusted to ensure computing time complexity is linear to number of individuals.

## CONCLUSION

For Mendelian (simple) traits, the Bayesian LASSO method outperforms gBLUP and sBLUP outperforms Bayesian LASSO in terms of prediction accuracy. For low heritability traits, which are challenging for both Bayesian LASSO and gBLUP, cBLUP is superior to both. Expanding the BLUP alphabet series with sBLUP and cBLUP enriches prediction method options so that the best method can be matched to the specific genetic architecture of a given trait of interest.

## Electronic supplementary material

Supplementary Material
