# Supplementary material for: Expanding the BLUP alphabet for genomic prediction adaptable to the genetic architectures of complex traits
Source: Heredity (Edinb). 2018 May 16;121(6):648–62. doi: 10.1038/s41437-018-0075-0 (PMC6221880; doi:10.1038/s41437-018-0075-0)
Supplement: Supplementary file 1 — Supplementary Material [file 41437_2018_75_MOESM1_ESM.pdf]

Table S1. Genotype and phenotype properties of real datasets.

Species	Sample size (N)	No. markers (M)	M/N ratio	Available real traits	Phenotype simulation
**Arabidopsis**	199	216,131	1086.09	81	No
**Mice**	1940	12,227	6.30	41	Yes
**Maize**	279	51,182	183.45	35	No
**Rice**	374	842,474	2252.60	1	Yes

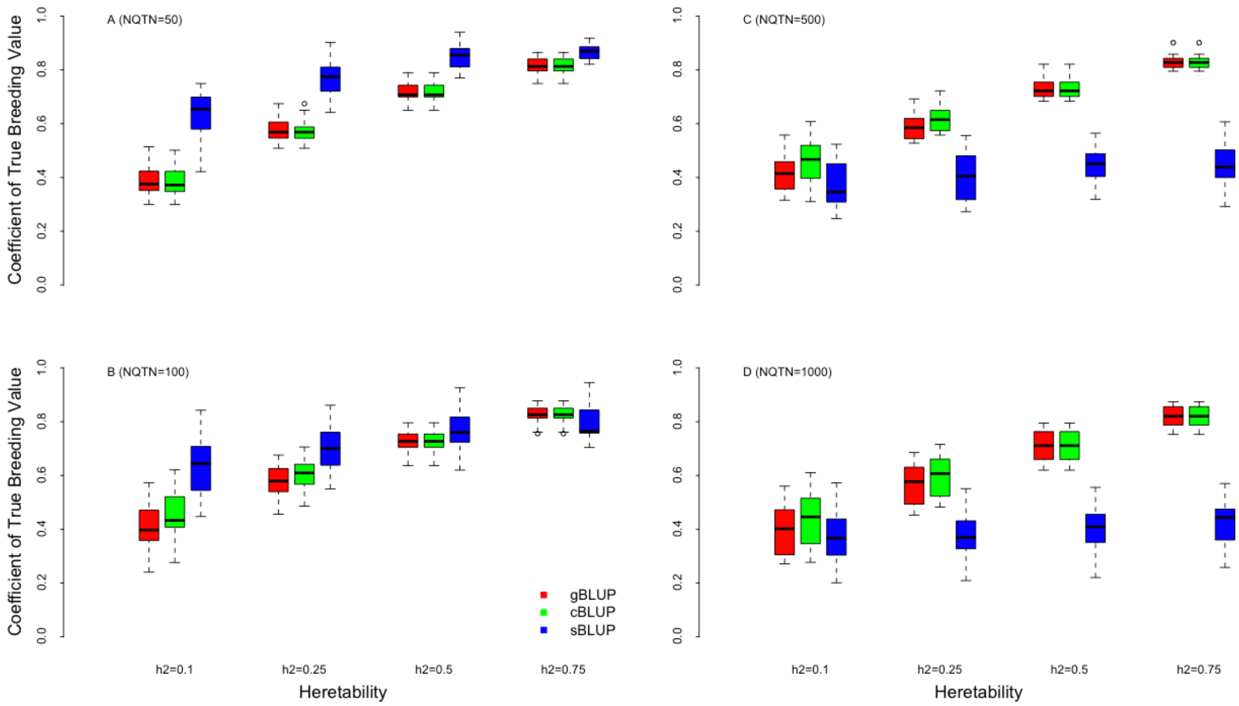

Figure S1. Regression coefficients of predicted breeding values over true breeding values by using three genomic prediction methods. We evaluated three prediction methods: conventional genomic Best Linear Unbiased Prediction (gBLUP), compressed BLUP (cBLUP), and SUPER BLUP (sBLUP). The comparisons were conducted on a simulated trait controlled by 50, 100, 500, and 1,000 Quantitative Trait Nucleotides (QTNs) under different heritability values (0.1, 0.25, 0.5, 0.75). These QTNs were randomly selected from a total of 12,227 available markers on 1,940 mice individuals from the WTCHG dataset. The predicted breeding values of the 1,940 individuals were evaluated in a five-fold cross-validation scheme. Regression coefficients were calculated as the predicted breeding values over the true breeding values for the individuals in the inference group. The process was replicated 20 times. Distributions of the regression coefficients demonstrate inflation of the predicted breeding values. The ideal prediction will have a regression coefficient of 1; however, shrinkage of BLUP as it approaches zero will reduce the regression coefficient. The farther the regression coefficient is from one, the more deflation.

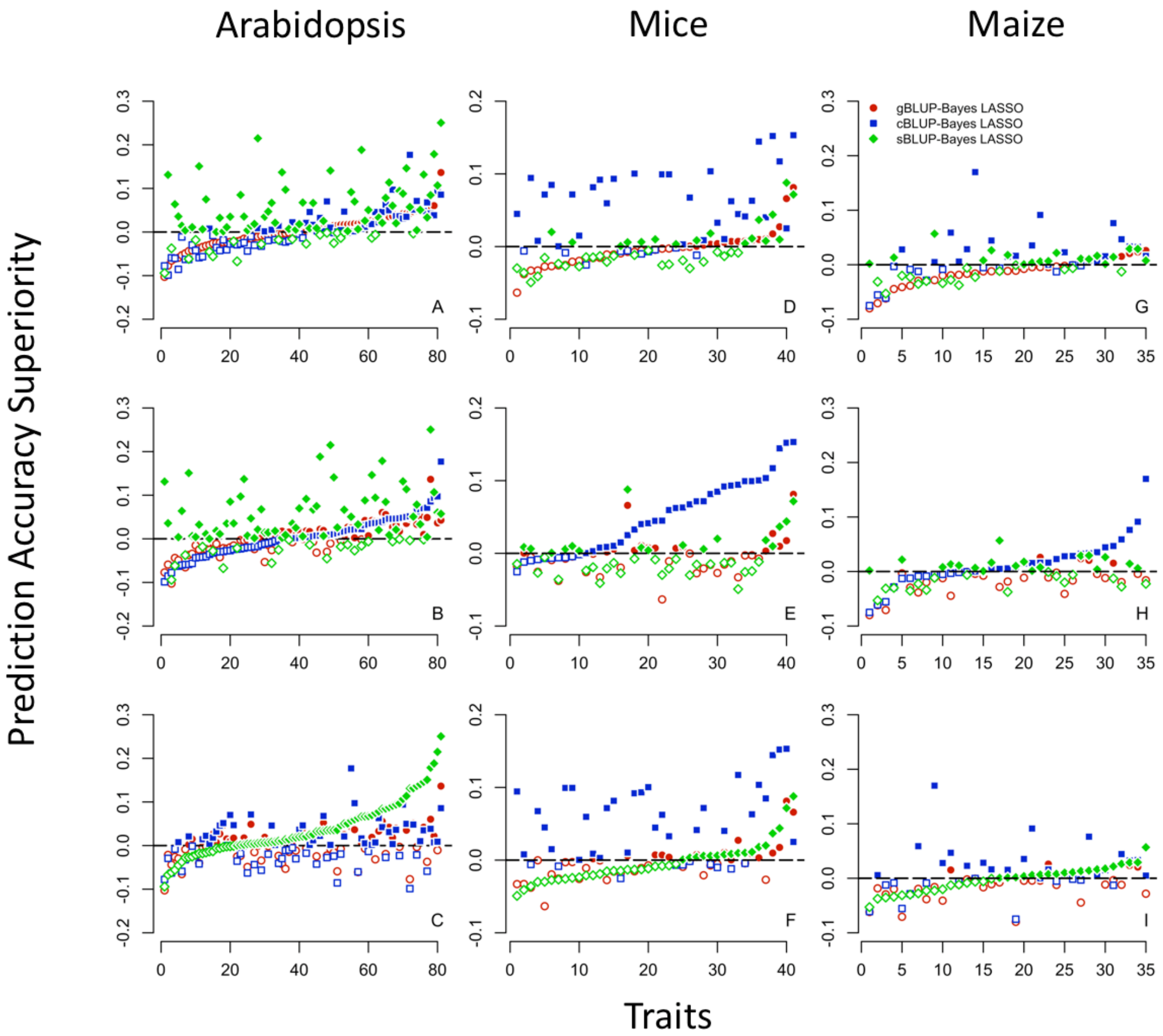

Figure S2. Sorted superiority of BLUP methods over Bayesian LASSO on real traits. The prediction accuracies of three BLUP methods (gBLUP, cBLUP, and sBLUP) were compared with Bayes LASSO. Superiority was calculated as the prediction accuracy difference between each BLUP method and Bayes LASSO. Traits for Arabidopsis, mice, and maize are presented as dots and sorted on the superiorities of gBLUP (top panel: **A**, **D,** and **G**), cBLUP (horizontal middle panel: **B**, **E,** and **H**), and sBLUP (bottom panel: **C**, **F,** and **I**). A dot is filled solid if a BLUP method is superior to Bayesian LASSO; otherwise, a dot is outlined. Prediction accuracies were evaluated using five-fold cross-validation. The cross-validations were replicated 40 times when gBLUP, cBLUP, and sBLUP were used and 20 times when Bayes LASSO was used. The first 80,000 iterations were used as "burn in" and the next 60,000 iterations were used to derive Bayesian estimations. We evaluated 81, 41, and 35 real traits for Arabidopsis (left panel: **A** to **C**), mice (vertical middle panel: **D** to **F**), and maize (right panel: **G** to **I**), respectively. sBLUP outperformed Bayesian LASSO on two-thirds of the traits in Arabidopsis (**C**). cBLUP outperformed Bayesian LASSO on two-thirds of the traits in mice (**E**). We found no clear trend in maize.

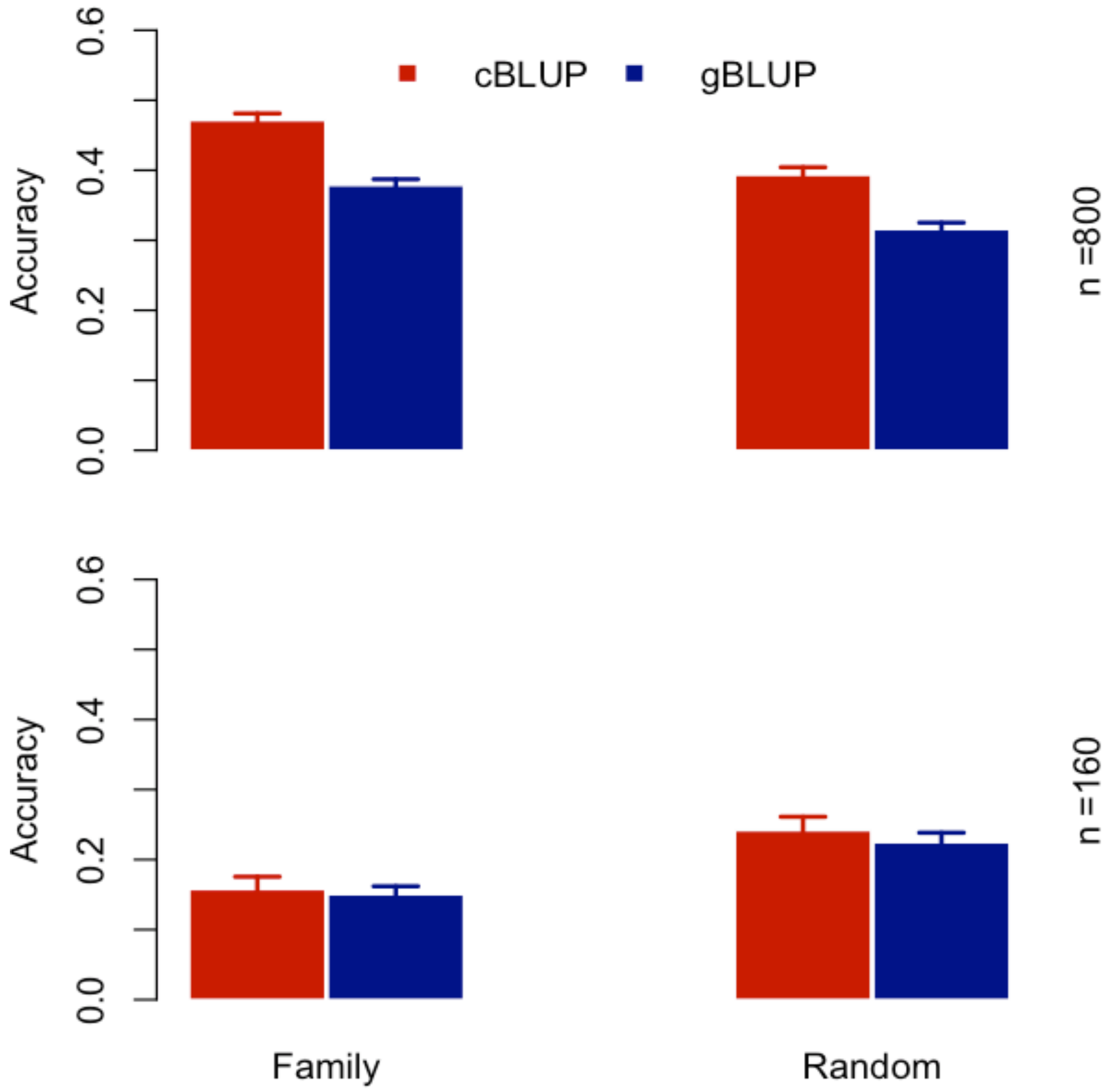

Figure S3. Comparisons between gBLUP and cBLUP under different levels of relatedness. The comparisons were conducted on a real trait named weight growth intercept from the WTCHG dataset. The dataset contained 1,940 mice individuals genotyped with 12,227 markers. These individuals were from 160 families (female parents). The levels of relatedness were created by different sampling schemes (family vs. random) and sample sizes (160 vs. 800). Under the family scheme, each family contributed one or five individuals to make samples with 160 or 800 individuals, respectively. Under the random scheme, the 160 or 800 individuals were randomly sampled from the 1,940 individuals regardless of family relationship. The predictions, using the 12,227 markers, were conducted by two methods, conventional genomic Best Linear Unbiased Prediction (gBLUP) and compressed BLUP (cBLUP). Prediction accuracy was evaluated through five-fold cross-validation with 20 replicates.

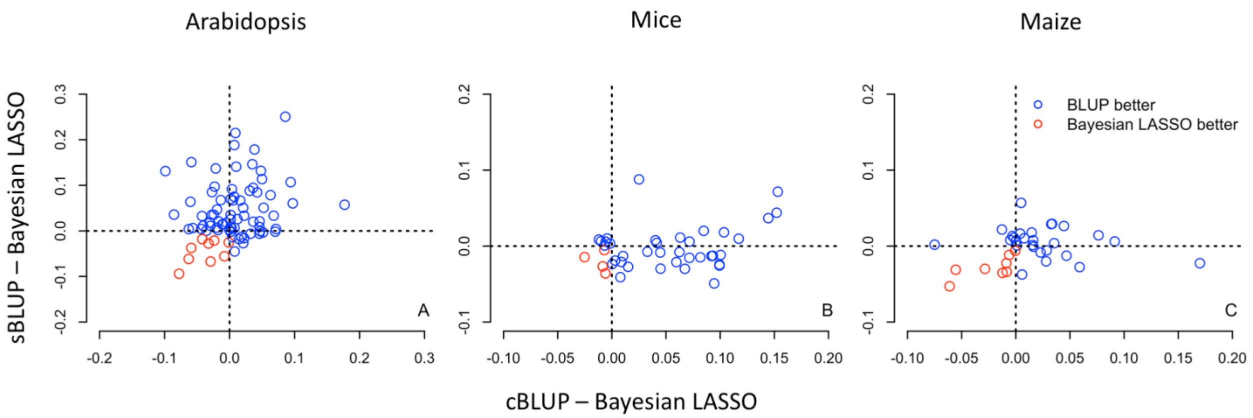

Figure S4. Superiority of BLUP methods over Bayesian LASSO in three species. Superiority was calculated as the genomic prediction accuracy difference between two new BLUP methods and Bayes LASSO. Superiority of cBLUP is illustrated as the horizontal axis and superiority of sBLUP as the vertical axis. The dashed lines indicate a difference of zero. Each circle represents a trait. In total, we evaluated 81, 41, and 35 traits for Arabidopsis, mice, and maize, respectively. A trait is colored red if Bayesian LASSO was superior to both sBLUP and cBLUP; otherwise, a trait is colored blue. Among the 157 traits, we found only 21 traits (13%) for which Bayesian LASSO outperformed sBLUP and cBLUP.
